# Therapeutic Role of Heterocyclic Compounds in Neurodegenerative Diseases: Insights from Alzheimer’s and Parkinson’s Diseases

**DOI:** 10.3390/neurolint17020026

**Published:** 2025-02-07

**Authors:** Nidhi Puranik, Minseok Song

**Affiliations:** Department of Life Sciences, Yeungnam University, Gyeongsan 38541, Republic of Korea

**Keywords:** Parkinson’s disease, Alzheimer’s disease, heterocyclic compounds, therapeutic targets, neurodegenerative diseases

## Abstract

Alzheimer’s and Parkinson’s are the most common neurodegenerative diseases (NDDs). The development of aberrant protein aggregates and the progressive and permanent loss of neurons are the major characteristic features of these disorders. Although the precise mechanisms causing Alzheimer’s disease (AD) and Parkinson’s disease (PD) are still unknown, there is a wealth of evidence suggesting that misfolded proteins, accumulation of misfolded proteins, dysfunction of neuroreceptors and mitochondria, dysregulation of enzymes, and the release of neurotransmitters significantly influence the pathophysiology of these diseases. There is no effective protective medicine or therapy available even with the availability of numerous medications. There is an urgent need to create new and powerful bioactive compounds since the number of people with NDDs is rising globally. Heterocyclic compounds have consistently played a pivotal role in drug discovery due to their exceptional pharmaceutical properties. Many clinically approved drugs, such as galantamine hydrobromide, donepezil hydrochloride, memantine hydrochloride, and opicapone, feature heterocyclic cores. As these heterocyclic compounds have exceptional therapeutic potential, heterocycles are an intriguing research topic for the development of new effective therapeutic drugs for PD and AD. This review aims to provide current insights into the development and potential use of heterocyclic compounds targeting diverse therapeutic targets to manage and potentially treat patients with AD and PD.

## 1. Introduction

A broad spectrum of neurological conditions characterized by alterations in the composition and operation of the central nervous system (CNS) are referred to as neurodegenerative diseases (NDDs). Parkinson’s disease (PD) and Alzheimer’s disease (AD) are the two most common NDDs. The main characteristic of these diseases is the massive deposition of misfolded protein aggregates consequent to aberrant production or overexpression of specific proteins [1,2]. In AD, the most prevalent cause of dementia worldwide, amyloid-β (Aβ) plaques and tau tangles are common hallmarks. The second most widespread protein misfolding disorder associated with dementia involves Lewy body (LB) pathology. This condition is defined by the intracellular aggregation of misfolded α-synuclein (α-syn) within cells, forming Lewy bodies and Lewy dystrophic neurites. These abnormalities are linked to disorders like dementia with Lewy bodies (DLB) and PD, collectively known as Lewy body disease (LBD). AD is a progressive NDD that affects many people worldwide [3,4], characterized by declining memory and cognitive dysfunction [5], and is the most prevalent type of dementia. The loss of synapses, the build-up of hyperphosphorylated tau protein inside cells, and the existence of extracellular Aβ peptide plaques are some of the main characteristics of AD [6]. Biochemical studies, along with research on transplanted neurons in PD patients and investigations using cell and animal models, suggest that the abnormal aggregation of α-synuclein (α-syn) and the spreading of pathology between the gut, brainstem, and higher brain regions may play a crucial role in the onset and progression of PD [7]. This condition is characterized by a progressive asymmetric slowness of movement (bradykinesia), rigidity, tremors, and gait disturbances, which occur alongside neuronal loss and the formation of α-syn-rich protein aggregates in the substantia nigra, referred to as Lewy bodies and Lewy neurites [8]. PD has both motor and nonmotor symptoms; whereas motor symptoms include tremors, resting tremors, bradykinesia, and stiffness, which are linked to a deficiency of dopamine in the striatum, nonmotor symptoms encompass sleep disorders, feelings of sadness, and cognitive impairments [9,10].

**Figure 1:**
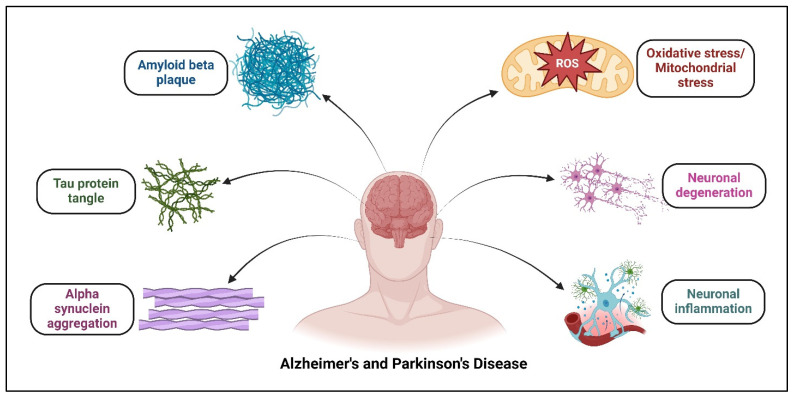
Pathogenic factors of Alzheimer’s disease and Parkinson’s disease. These factors include oxidative stress/mitochondrial stress, neuroinflammation and neurodegeneration, and plaques of insoluble proteins including Aβ, tau, and α-syn.

The pathogeneses of AD and PD share common pathways of degeneration. The most common etiological factors include oxidative stress (OS), mitochondrial stress, neuroinflammation, neurodegeneration, and the build-up of insoluble proteins, as shown in Figure 1.

## 2. Therapeutic Targets in Alzheimer’s and Parkinson’s Disease

The exact etiologies of AD and PD are still unknown, and there is not a safe and efficient treatment to stop these diseases’ progression or cure them [11,12]. A recent review article highlights the ongoing drug trials for AD and mentions that 187 trials are currently underway to evaluate 141 AD drugs, with over 57,000 participants [13], while another article for PD identified 145 registered and ongoing clinical trials [14]. For safe and effective treatment development, the well-detailed pathogenesis needs to be understood first. The primary causes of AD pathogenesis include tau NFTs, Aβ plaques, neuroinflammation, OS, cholinergic dysfunction, glutamate excitotoxicity, and alterations in neurotrophin levels [15,16,17,18,19,20,21]. These pathogenic factors are the possible major therapeutic targets for AD treatment and management [18,19,21,22,23,24,25].

**Figure 2:**
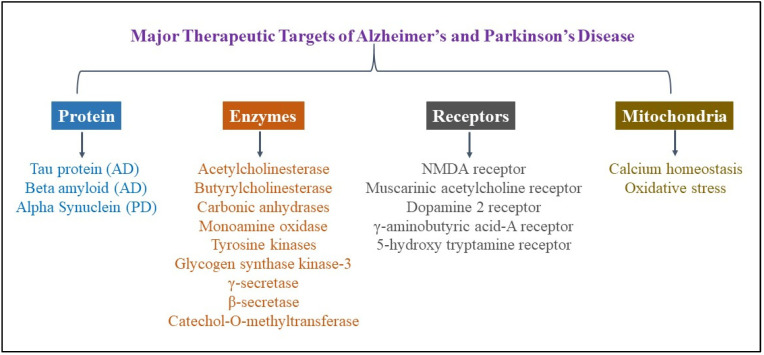
Major therapeutic targets of Alzheimer’s and Parkinson’s disease. As AD and PD share major common etiological factors, they also share some therapeutic targets, which mostly include proteins, enzymes, receptors, and mitochondrial/oxidative stress.

PD is a multifactorial disorder with similar phenotypes. According to recent research studies, classifying patients based on genes involved in PD could be the most practical approach, as clinical phenotypes or biomarkers may not accurately represent the disorder. Mutations are common genetic causes of PD, with patients with these variants having varying clinical characteristics and pathologies [26]. Several autosomal dominant and autosomal recessive genes have been identified, with *SNCA* (Synuclein Alpha), *LRRK2* (Leucine-Rich Repeat Kinase 2), *PRKN* (Parkin RBR E3 Ubiquitin Protein Ligase), *PINK1* (PTEN-induced kinase 1), and *GBA1* (glucosylceramidase beta 1) being the most commonly linked genes associated with PD pathology [12]. α-Syn aggregates are Lewy bodies’ primary components, which are PD’s pathological hallmarks [27]. Experimental evidence suggested that a decrease in glucocerebrosidase activity is associated with the accumulation of α-syn [28]. Similarly, a decrease in the TMEM175 (Transmembrane protein 175) level causes the accumulation of α-syn in neurons, leading to the loss of dopaminergic neurons [29]. The PRKN gene encodes the parkin protein that preferentially protects dopaminergic neurons from mitochondrial stress, and loss of this exacerbates mitochondrial dysfunction in neurons [30]. The PINK1/parkin pathway has been shown to significantly enhance the function of PINK1 and parkin, a crucial process in mitophagy essential for maintaining mitochondrial health, and the most potent therapeutic target for PD [31]. Several recent preclinical and clinical candidates are being developed to target various proteins and receptors associated with NDDs, as listed in Figure 2. These potential targets include tau protein [32,33,34,35], amyloid beta [36,37,38,39], α-syn [40,41], acetylcholinesterase (AChE) [42,43,44,45,46], butyrylcholinesterase (BChE) [47,48], tyrosine kinases [49], glycogen synthase kinase-3 [50,51,52], γ- and β-secretases [53,54], monoamine oxidase (MAO) [55], N-methyl-D-aspartate (NMDA) receptors [56,57,58], muscarinic acetylcholine receptors [59,60], dopamine D2 receptors [61,62], GABA-A receptors [63,64], the 5-hydroxytryptamine (5-HT6) receptor [65], the OS pathway, and mitochondrial dysfunction [66,67,68,69]. Major therapeutic targets are discussed briefly one by one.

Medications aimed at alleviating dementia symptoms fall into two main categories: those that address cognitive decline and those that target behavioral and psychological symptoms of dementia (BPSD). BPSD mitigators include suvorexant, an orexin receptor antagonist for sleep disorders, and brexpiprazole, an atypical antipsychotic suitable for treating agitation in dementia patients with its once-daily dosing. Examples of cognitive decline mitigators include glutamate inhibitors, like memantine, and cholinesterase inhibitors, such as donepezil, rivastigmine, and galantamine [70].

**Figure 3:**
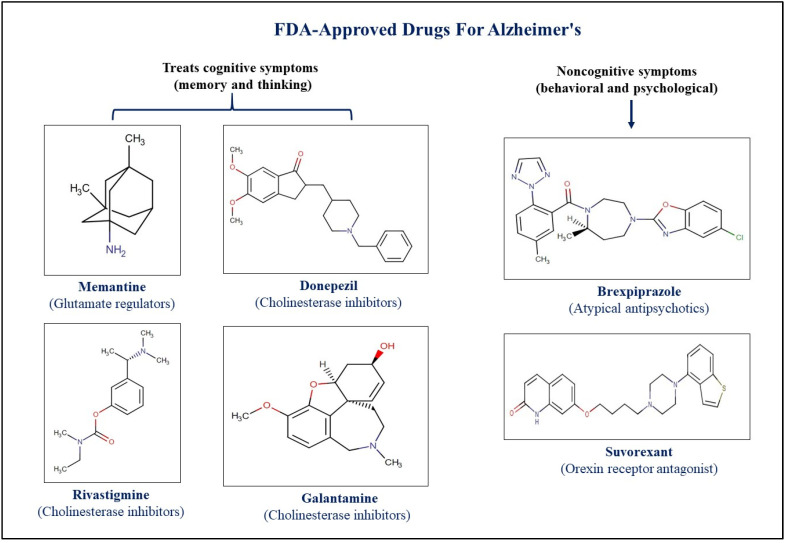
FDA-approved drugs for treatment and management of Alzheimer’s disease.

**Figure 4:**
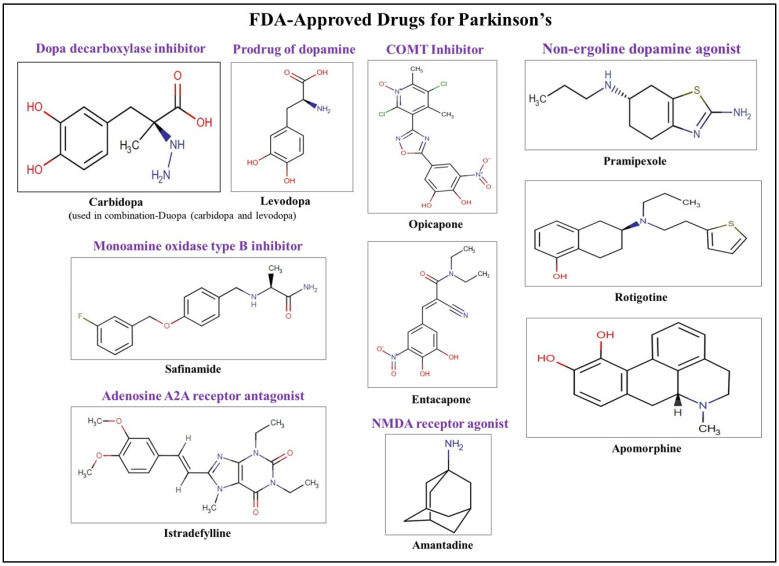
FDA-approved drugs for treatment and management of Parkinson’s disease.

Pharmaceuticals that may slow down the clinical decline of AD patients and treatments that may temporarily relieve some of the disease’s related symptoms are the two types of medications approved by the US Food and Drug Administration (FDA) [71]. Figure 3 and Figure 4 illustrate the FDA-approved therapeutic drugs for AD and PD, respectively. While several therapies have received FDA approval to alleviate symptoms, none can slow, stop, or reverse the progression of these diseases. The primary constraint of these symptomatic treatments is the failure to address the underlying disease progression. AChE inhibitors, such as Razadyne, Aricept, and Exelon, are used in the management of dementia and cognitive decline symptoms. These inhibitors facilitate neuronal transmission and cognitive function by maintaining the levels of acetylcholine (ACh) in synaptic gaps and preventing AChE from degrading it [72]. Moreover, memantine hydrochloride (Namenda), an NMDA receptor antagonist, aims to prevent excess glutamate from overstimulating neurons and causing excitotoxic damage. Additionally, Belsomra, a dual orexin receptor antagonist originally used for insomnia, is available to address sleep disorders associated with AD [72]. All these FDA-approved drugs are heterocyclic, and their therapeutic categories, targets, and applications are summarized in Table 1.

Drugs like Sinemet, Paracopa, Rytary, and Duopa which contain levodopa and carbidopa are the most widely used approved treatments for PD motor symptoms. Carbidopa, a dopa decarboxylase inhibitor, decreases levodopa’s extracerebral metabolism before it crosses the protective blood–brain barrier, increasing the neurotransmitter’s brain bioavailability. Levodopa is a dopamine precursor. COMT inhibitors, which include Comtan, Tasmar, and Ogentys, are another family of FDA-approved therapies. These drugs have shown promise when used with carbidopa/levodopa therapy. By blocking the COMT enzyme, these medications stop levodopa from being broken down extracerebrally and raise its plasma levels. By attaching to dopamine receptors, dopamine agonists can penetrate the blood–brain barrier and replicate the effects of dopamine, hence lowering dyskinesia. Levodopa dosage requirements are decreased with carbidopa and COMT inhibitors. Currently, Mirapex, Requip, Apokyn, Kynmobi, and Neupro are FDA-approved medications that function as dopamine agonists. In the brain, the enzyme MAO-B degrades dopamine. Eldepryl, Zelapar, Azilect, and Xadago are examples of MAO-B inhibitors that have been approved by the FDA. The FDA has approved NMDA receptor antagonists such as Osmolex, Gocovri, and Symmetrel to treat dyskinesia. In PD patients, adenosine 2A antagonists like Nourianz exhibit neuroprotective effects. Anticholinergic medications, like Cogentin and Artane, have been licensed for the treatment of PD patients’ tremors by balancing the imbalance between dopamine and ACh. It is important to remember that every approved treatment is symptomatic [72].

### 2.1. Proteins

#### 2.1.1. Aggregated Protein

The amyloid peptide is a major component of amyloid plaques and has been implicated in numerous studies as a major pathogenic factor of AD. Tau protein (another important histopathological characteristic of AD) clumps inside neurons make up the majority of NFTs [73].

Multiple locations of tau phosphorylation regulate the protein’s ability to connect to microtubules; hyperphosphorylation, on the other hand, causes AD pathogenesis. According to research, tau impairment is most likely the effector molecule of neurodegeneration, and Aβ may start the chain of events leading to tau hyperphosphorylation. By mediating the activation of several pathways, Aβ speeds up the hyperphosphorylation of tau and exacerbates tau-induced neurotoxicity, which results in AD [74]. More significantly, it has now been demonstrated that downregulating tau partially restores transcriptional perturbations and that Aβ and tau cooperate to hinder the transcription of genes implicated in synaptic function [75,76]. Recent research has shown links between abnormal tau and Aβ protein behavior and several neurological conditions, including AD, DPD, and ALS, as well as retinal neurodegenerative conditions, including age-related macular degeneration and glaucoma [77].

To investigate the possible molecular routes through which tau may contribute to the pathogenesis of PD, several studies have used transgenic PD mouse lines in which tau is altered, decreased, or removed [78]. α-Syn is predominantly found at the presynaptic terminals and associated with synaptic vesicles [79]. α-Syn exhibits a range of species, including monomers, oligomers, and fibrils, each of which has distinct neurotoxic characteristics [80]. One of the main molecular mechanisms behind the pathophysiology of PD is the aggregation of α-syn. These aggregates, which disturb cellular homeostasis and contribute to neurodegeneration, can form at synaptic terminals and are frequently caused by a breakdown in proteostatic defenses or aberrant interactions between α-syn and lipids [81,82]. PD patients’ autopsies also showed that tau and α-syn were colocalized in LBs. Tau interacts with α-syn and affects the pathogenesis of α-syn in Parkinson’s disease, according to experimental studies [78]. Tau’s interaction with α-syn and the acceleration of its aggregation were demonstrated experimentally in a recent study. Tau-modified α-syn fibrils exhibit increased seeding activity in comparison to pure α-syn fibrils, causing neurotoxicity, synaptic impairment, and mitochondrial dysfunction in vitro. Compared to mice injected with pure α-syn fibrils, mice injected with tau-modified α-syn fibrils in the striatum experience more severe α-syn disease, motor dysfunction, and cognitive impairment. Both in mice injected with α-syn fibrils and α-syn A53T transgenic animals, tau knockout reduces the spread of α-syn pathology and PD-like symptoms. To sum up, tau promotes α-syn aggregation and spread in PD [83]. Therefore, to improve therapeutic efficacy, it is essential to understand the molecular properties of pathogenic proteins deposited in each non-AD NDD [84].

#### 2.1.2. Dopamine Transporter (DAT)

The dopamine transporter (DAT) is a transmembrane protein that belongs to the Na^+^/Cl^−^-dependent neurotransmitter transporter family. It consists of 12 transmembrane helices and undergoes conformational changes upon ligand binding. These structural changes are essential for facilitating the translocation of dopamine into the neuron [85]. DAT facilitates the transport of extracellular dopamine into the intracellular space, playing a crucial role in regulating dopamine neurotransmission. A reduction in DAT density is implicated in PD, and, simultaneously, dopamine turnover is elevated in early symptomatic PD [85,86]. Recent studies have revealed that blocking DAT shows the potential to increase dopamine levels and slow disease progression, highlighting its therapeutic promise [87]. Nigrostriatal dopaminergic dysfunction contributes to parkinsonism and cognition independently of extranigral cortical thinning in patients with AD [88].

### 2.2. Enzymes

#### 2.2.1. Cholinesterase Enzymes

Cholinesterases are crucial enzymes found in both the central and peripheral nervous systems. These enzymes facilitate neurotransmission by enabling the conduction of nerve impulses at cholinergic synapses. The two main forms of cholinesterases are AChE and BChE. BChE, closely related to AChE, acts as a co-regulator of cholinergic neurotransmission by hydrolyzing ACh. Research has shown that during the progression of AD, there is an increase in BChE activity in the most affected brain regions, such as the temporal cortex and hippocampus. This heightened BChE activity is also significant in the early stages of senile plaque development, particularly in relation to Aβ aggregation. Consequently, inhibiting both AChE and BChE has been recognized as an important strategy for effectively managing AD, as it leads to increased availability of ACh in the brain and a reduction in Aβ deposition. One common method for treating a variety of mental illnesses is the inhibition of the AChE and BChE enzymes. Cholinesterase inhibitors are used in this context to alleviate the symptoms of neurological conditions such as AD and dementia. The role of several heterocyclic scaffolds comprising N, O, and S in the design and development of novel potential AChE and BChE inhibitors to treat AD has been investigated in recent years. Additionally, a thorough structure–activity relationship (SAR) has been developed for the potential future development of new medications to treat AD. New powerful cholinesterase inhibitors have been designed using the majority of heterocyclic motifs [89]. The oldest and least expensive antiparkinsonian medicine is an anticholinergic agent used to treat PD [90].

#### 2.2.2. Carbonic Anhydrases (CAs)

CAs, metalloenzymes that reversibly hydrate carbon dioxide, play an antioxidant role in cells during oxidative processes. They are crucial in brain pH control, neuronal excitability, and cognition and can impact animal learning. Moreover, CA II is associated with abundant plaque proteins, suggesting a central role in plaque development. Inhibiting mitochondrial CAs may slow NDD progression [91]. Mitochondrial proteomic profiling reveals increased CA II in aging and neurodegeneration, suggesting that targeting CA II associated with mitochondria could specifically modulate age-related impairments and neurological diseases [92]. Recent research indicates that CA activation may be a viable therapeutic strategy for the treatment of AD since it is implicated in brain functions necessary for the transmission of neuronal messages [93] and various illnesses that are marked by cognitive issues and memory loss. Certain substances that can activate CAs have been discovered and suggested as a potential solution to neurodegenerative issues [94].

#### 2.2.3. Monoamine Oxidase Enzyme (MAO)

MAO, an enzyme anchored to the mitochondrial membrane, catalyzes the oxidative deamination of both endogenous and exogenous monoamines, including key neurotransmitters such as dopamine, serotonin, and adrenaline. Recent research has highlighted MAO as a promising therapeutic target for NDDs, as its role in neurodegeneration has been increasingly recognized [95,96,97]. The MAO enzyme, present in both the brain and peripheral tissues, plays a crucial role in regulating neurotransmitter levels by deaminating biogenic amines. MAO exists in two isoforms, MAO-A and MAO-B, which are differentiated by their three-dimensional structures, substrate preferences, inhibitor selectivity, and amino acid sequences [55,98]. Both isoforms of MAO and their inhibitors, such as clorgiline and selegiline—commonly prescribed for neurological and neurodegenerative disorders—interact with substrates like dopamine, tyramine, and tryptamine. MAO-A inhibitors are primarily utilized for treating depression, while MAO-B inhibitors are widely used in managing PD. Additionally, the potential efficacy of MAO-B inhibitors in treating AD is an area of ongoing research [55].

Activated MAO facilitates two consecutive cleavages of APP by β-secretase and γ-secretase, leading to the aggregation of Aβ. Additionally, active MAO exacerbates cholinergic neuronal damage and dysfunction in the cholinergic system, contributing to the formation of NFTs and subsequent cognitive decline. The overall anti-AD effect of MAO inhibition is primarily attributed to the reduction in OS induced by MAO enzymes [99] and neurochemicals like dopamine, serotonin, and adrenaline [100].

MAO inhibitors have recently been developed using a variety of structural scaffolds, including chalcones, conjugated dienones, isatins, chromones, coumarins, pyrazolines, quinazolines, β-carbolines, and compounds generated from benzyloxy [100,101,102].

#### 2.2.4. Catechol-O-Methyltransferase (COMT) Inhibitors

COMT is an enzyme that catalyzes the transfer of a methyl group from S-adenosylmethionine and degrades catecholamines. COMT regulates dopamine metabolism in the prefrontal cortex, affecting neuropsychiatric disorders and cognitive disruptions. It plays a crucial role in regulating prefrontal levels, affecting both the CNS and peripheral regions [103]. The two primary isoforms of the COMT protein are soluble (S-COMT) and membrane-bound (MB-COMT). In the brain, MB-COMT is the predominant kind, whereas the S-COMT isoform is more common in other organs. Through precise dopamine flux modulation, COMT plays a critical role in the prefrontal cortex’s and striatum’s dopamine degradation, which impacts cognition and cognitive control [104]. COMT inhibitors function by preventing catechols, such as dopamine, from degrading into inactive chemicals. This reduces the symptoms of CNS diseases by making L-dopa available to the brain [105]. Through its effects on the metabolism of catecholamine neurotransmitters and estrogen, COMT is emerging as a key player in the pathogenesis of AD. A subgroup of AD patients has been found to have impaired executive functioning, which is linked to a more severe pathology, a faster rate of disease development, and a lower survival rate [106].

**Figure 5:**
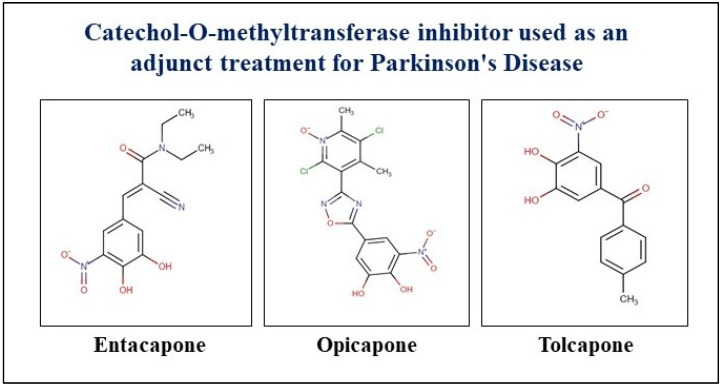
Chemical structure of COMT inhibitors entacapone, opicapone, and tolcapone used in PD treatment and management.

COMT inhibitors are among the recommended first-line therapies to be combined with levodopa for managing end-of-dose motor fluctuations in patients with advanced PD [107]. The three most important COMT inhibitors are entacapone, tolcapone, and opicapone used in PD treatment and management, as represented in Figure 5 [108].

### 2.3. Mitochondrial Calcium Homeostasis and Oxidative Stress

Mitochondria are a hub for cellular communication and metabolism. In addition to being necessary for a variety of mitochondrial processes, ions like Ca^2+^ and iron can be stored within the mitochondria to preserve cellular ionic equilibrium. Ion intake, transit, and storage are coordinated by ion channels and transporters in the mitochondrial and plasma membranes. Via voltage- or ligand-gated Ca^2+^ channels located throughout the cell surface, Ca^2+^ enters the cell. Iron is transported by endocytosis from iron-bound carrier proteins like transferrin, and internal iron can be absorbed by mitochondria through a number of IMM transporters. Even though iron and Ca^2+^ homeostasis disruptions have been documented separately in PD and AD models, their molecular relationship and roles in disease vulnerability are still unclear [109].

A complex web of interrelated factors, including high metabolic activity, neurotransmitter autoxidation, elevated redox-active transition metal content, modest antioxidant defense, glutamate excitotoxicity, and altered Ca^2+^ influx and related signaling processes, make the brain especially vulnerable to oxidative insults [110,111]. Redox balance changes and corresponding changes in redox-sensitive signaling pathways can be caused by a compromised antioxidant system or by abnormal and persistent free radical production. Although brain ROS and RNS are second messengers engaged in intracellular signaling at the normal level, elevated free radical levels have detrimental effects on biological macromolecules that are linked to the pathophysiology of NDDs and the aging process [112,113]. Although the precise mechanisms behind the etiopathogenesis of AD and PD are still unknown, there is a wealth of evidence suggesting that the pathophysiology of these neurological disorders is significantly influenced by the excessive production of ROS and RNS, as well as by a depleted antioxidant system, mitochondrial dysfunction, and intracellular Ca^2+^ dyshomeostasis. The incidence of age-related NDDs has dramatically grown as life expectancy has increased. However, only very limited palliative care is accessible, and no effective protective medicine or therapy is available. Therefore, the development of disease-modifying treatments and preventive measures to treat AD/PD is urgently needed. Since oxidative damage and neuropathology in these disorders are caused by dysregulated Ca^2+^ metabolism, finding or creating substances that can restore Ca^2+^ homeostasis and signaling could offer a neuroprotective therapy option for NDDs [114].

According to preclinical research, controlling calcium levels may help in both in vitro and in vivo models of AD by directly blocking calcium channels or calcium-dependent downstream cascades. Calcium channel blockers, either alone or in combination with antioxidants, GSK-3 inhibitors, and cholinesterase inhibitors, have been a part of the majority of effective models to date. However, in both acute and chronic settings, it has also been demonstrated that activating specific calcium channels, including TRPV1, TRPC6, and TRPML1, has positive effects. Thus, the molecular target and its corresponding cellular physiological role, the afflicted brain circuit, and the stage of AD pathology should all be taken into account when adjusting intracellular calcium levels. For instance, the hippocampus’s synaptic plasticity processes depend on a homeostatic rise in calcium levels, but an overabundance of these levels can cause late-onset neurodegeneration, excitotoxicity, and glial-induced neuroinflammation problems across the board [115].

The study of mitochondria-targeted medication has gained attention concerning NDDs, PD, and AD. It is well accepted that the pathophysiology of PD and other related NDDs is significantly influenced by mitochondrial dysfunction [116]. However, intricate genetic control governs mitochondrial malfunction. There is mounting evidence that genes linked to Parkinson’s disease either directly or indirectly impact mitochondrial integrity. As a result, there are several clinical opportunities for tailored modulation of mitochondrial function in the therapy of PD [117]. Disturbances in iron and calcium homeostasis and accumulation are strongly linked to pathological features of PD, including intracellular α-syn deposition and dopaminergic neuronal death [118]. Several NDDs associated with OS have been documented, including PD, AD, spinocerebellar ataxia, ALS, and HD [119].

## 3. Therapeutic Potential of Heterocyclic Compounds in AD and PD Management

In many active medications and natural compounds, heteroatoms and heterocyclic scaffolds are commonly found as fundamental components. More than 85% of all physiologically active chemicals are heterocycles or contain a heterocycle, with nitrogen heterocycles being the most common backbone in their complex structures. These statistics reveal and highlight how important heterocycles are to contemporary medication design and discovery [120].

Heterocycles are important in medicinal chemistry and pharmaceutical industries because of their various actions and stability with different substituents. Several diseases such as cancer, AIDS, CNS disorders, CVDs, and diabetes involve the usage of drugs with heterocyclic ring systems as their major pharmacophore [121]. In essence, the ring structures of heterocycles are made up of atoms other than carbon, with oxygen, nitrogen, and sulfur serving as the most common substituents. Heterocycles can be categorized as oxygen-, nitrogen-, or sulfur-based based on the heteroatom or atoms that are present in the ring structures. Within each class, compounds are arranged according to the size of the ring structure, which is defined by the total number of atoms [122].

Numerous lead compounds with CNS action have been developed with success using polycyclic cage scaffolds. Polycyclic cage derivatives can affect a number of NDDs, including drug misuse, schizophrenia, stroke, PD, HD, and AD. When creating therapeutically active medicines to treat neurological illnesses, these cage moieties—which include derivatives of adamantane and pentacycloundecane—improve the pharmacokinetic and pharmacodynamic characteristics of conjugated parent medications [123].

**Figure 6:**
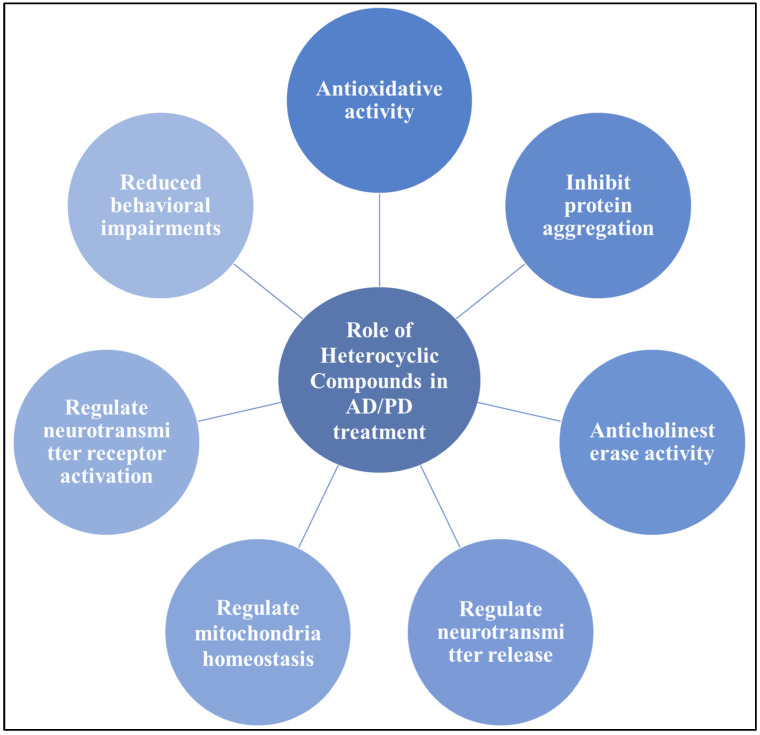
General overview of the significant roles of heterocyclic compounds in the treatment of Alzheimer’s disease and Parkinson’s disease. Various heterocyclic motifs exhibit diverse activities, including anticholinesterase activity, antioxidant properties, inhibition of protein aggregation, regulation of neurotransmitter release in synapses, maintenance of mitochondrial homeostasis, modulation of neurotransmitter receptor activation, and reduction in behavioral impairments.

Heterocycles that include nitrogen are a significant and distinct class of molecules that are used extensively in organic chemistry. In medicinal chemistry, they are unique in that they are a valuable source of therapeutic compounds. Over 75% of FDA-approved medications that are presently on the market have heterocyclic moieties that contain nitrogen. N-heterocyclic compounds are widely found in nature, have a variety of physiological and pharmacological characteristics, and are building blocks of several molecules that are significant to biology. Vitamins, nucleic acids, medications, antibiotics, colors, and agrochemicals are also included [124]. S-heterocycles continue to be a crucial component of FDA-approved medications and medicinally active substances. Researchers’ focus has switched to other heterocycles, particularly S-heterocycles, as a result of the thorough investigation of nitrogen heterocycles in medicinal chemistry. In contrast to earlier N-heterocycles, numerous attempts have been made to synthesize a range of novel sulfur-containing molecules with a low toxicity profile and significant therapeutic utility. There have been reports in the past five years that highlight the importance and use of sulfur-containing heterocycles in the drug development process, including thiirane, thiophene, thiazole, thiopyran, and thiazolidine [125]. In organic chemistry, the oxygen-containing heterocycles constitute a significant class of molecules. In medical research, these substances—coumarin and oxazole—are utilized as medications [126]. Major therapeutic applications of heterocyclic motifs in AD/PD treatment are shown in Figure 6. Various studies show the potential role of synthetic heterocyclic compounds in AD/PD treatment is summarized in Table 2 and Table 3 providing a summary of in vitro studies showing the inhibitory impact of synthetic heterocyclic compounds on major therapeutic enzymatic targets of AD and PD.

### 3.1. Nitrogen-Based Heterocyclic Moieties

Nitrogenous heterocyclic moieties as the core nucleus and/or in combination as an antidepressant chemical include quinazoline, pyridine, pyrimidine, pyrrolidine, imidazole, pyrazole, piperidine, oxadiazole, benzimidazole, benzothiazole, piperazine, triazine, purine, benzoxazole, and isoxazole. These compounds with various structural characteristics have been shown to have antidepressant effects through a variety of methods, including COMT inhibitors, MAO inhibitors, and selective serotonin reuptake inhibitors (SSRIs) [127]. Currently, privileged structures such as benzopyrans, arylpiperazines, biphenyls, and indoles are recognized as valuable strategies. Various nitrogen- and oxygen-containing heterocycles, including pyrazoles, hydrazinylthiazoles, xanthones, coumarins, and chromones, have been extensively explored as scaffolds for developing new MAO-B inhibitors. Among these, nitrogen-based derivatives play a pivotal role, with numerous studies emphasizing hydrazines, thiazoles, and indoles as essential scaffolds for the design of novel MAO-B inhibitors [90].

As a nonaromatic heterocyclic nucleus, piperidine has a six-membered ring with one secondary amine group (−NH−) and five methylene groups (−CH_2_−). Among heterocyclic compounds, piperidines are well known for their several pharmacological uses, which include antipsychotic, antidepressant, and neuroprotective qualities. Many drugs, including clarinex, donezepil, raloxifene, and methylphenidate, have been found to have piperidine as their principal chromophore [128].

Piper nigrum, the plant from which black and white pepper grains are derived, contains the alkaloid piperine. It has been demonstrated that piperine exhibits a variety of activities, including MAO inhibitory action. An MAO-A and -B test was used to screen several compounds associated with piperine and antiepilepsirine, which may have been used in PD [129]. The main alkaloid in a family of structurally related substances present in Lobelia inflata is α-lobeline, also known as lobeline, a lipophilic, nonpyridino, alkaloidal component of Indian tobacco. By interacting with vesicular monoamine transporter 2, lobeline has been shown to suppress DA uptake into synaptic vesicles and promote the reverse transportation of DA from synaptic vesicles. Lobeline reduces behavioral impairments in rats and exhibits protective benefits against MPTP-induced dopaminergic neuron death [130].

As with methylphenidate analogs, its 3,4-dichloroaryl cousin is one of the more powerful benzoylpiperidines. Here, one study shows that these chemicals probably bind similarly at hDAT using homology models. The effectiveness of these hybrids may be influenced by the electronic nature of the substituents, as evidenced by the fact that the 3,4-dichlorobenzoylpiperidine analog of 1a is more potent than its 3,4-dimethyl equivalent. In addition, the 3,4-benz-fused (naphthyl) benzoylpiperidine analog functions at hDAT in the same way as its methylphenidate cousin. Similar to its methylphenidate analog, the naphthyl molecule also operates at the human serotonin transporter (hSERT), albeit in a somewhat different way and with less effectiveness than the other members of the two series. The benzoylpiperidines are a new structural class of hDAT reuptake inhibitors that work similarly to their methylphenidate equivalents [131].

In the Aβ(1-42) rat model of AD, one study examined the potential memory-boosting and antioxidant qualities of the methanolic extract of Piper nigrum L. fruits. In vivo methods were used to investigate the plant extract’s memory-boosting properties. Additionally, the total content of reduced glutathione, malondialdehyde, and protein carbonyl levels, as well as the activities of superoxide dismutase, catalase, and glutathione peroxidase, were used to evaluate the antioxidant activity in the hippocampus. Rats treated with Aβ(1-42) showed an increase in working memory and reference memory errors in the radial arm maze test and a decrease in the percentage of spontaneous alternations in the Y-maze task. The administration of the plant extract demonstrated antioxidant properties and markedly enhanced memory performance. Overall, the results suggest that the plant extract ameliorates Aβ(1-42)-induced spatial memory impairment by attenuating the OS in the rat hippocampus [132].

Both plants and animals naturally contain the heterocyclic aromatic ring system indole. It has electronic and steric properties that are advantageous for attaining bioavailability and pharmacological effects. It is composed of a six-membered benzene ring fused with a five-membered pyrrole ring. Because it may bind with different receptors and produce a wide range of biological functions, indole is known as a flexible pharmacophore. It is therefore regarded as one of the most advantageous frameworks in pharmaceutical chemistry [73]. Zhou L and colleagues developed, produced, and assessed a number of diosgenin–indole compounds for the potential therapy of AD. These compounds were tested for their neuroprotective effects against Aβ, 6-hydroxydopamine (6-OHDA), and hydrogen peroxide (H_2_O_2_) damages. The addition of an indole fragment and an electron-donating group at C-5 on the indole ring may have neuroprotective effects, according to preliminary structure–activity relationships. The most promising contender against cellular damage caused by H_2_O_2_, 6-OHDA, and Aβ1-42 was compound 5b, according to the results [133]. To treat AD, thirteen new oxathiolanyl, pyrazolyl, and pyrimidinyl indole derivatives were created and produced. The method was as follows: Both the AChE and BChE enzymes were tested in an in vitro enzyme assay. Furthermore, cytotoxicity on a normal cell line and antioxidant assay results were established. Dynamic simulations and molecular docking were used to validate the binding mode in the active regions of both esterases. Studies on toxicity, metabolism, excretion, distribution, and absorption were also conducted in silico. Superior inhibitory action against AChE and BChE was demonstrated by a few of them [134].

As demonstrated by its ranking first among the top five most prevalent five-membered nonaromatic nitrogen heterocycles and its presence in thirty-seven new molecular and/or first-in-class entities approved by the FDA, such as captopril, lincomycin, clemastin, and remoxipride, the pyrrolidine nucleus is one of the preferred scaffolds in pharmaceutical sciences and drug design. Many new pyrrolidine compounds have been created in recent decades for their potential in the CNS, and they have shown promise in treating neurodegenerative illnesses, mental illness, and other CNS problems [135,136]. Numerous pyrrolidine derivatives have been developed as conformationally limited analogs of profadol for analgesic action, and various N-(pyrrolidin-3-yl)-naphthamide analogs bind with high affinity for both the D2 and D3 dopamine receptor subtypes. In addition to these, several pyrrolidine-containing substances have been suggested over time to treat AD [135]. After preparing some new N-benzoylthiourea-pyrrolidine carboxylic acid derivatives with an imidazole moiety and assessing their diverse biological activities, it was discovered that the produced compounds may have inhibited AChE and BChE, the primary targets for AD [137].

Researchers have developed two novel hMAO-B inhibitors using benzimidazole as a scaffold and a primary amide group. The most potent inhibitor inhibits hMAO-B competitively and reversibly. The compound shows a good safety profile, ideal pharmacokinetic properties, and blood–brain barrier permeability. In a PD mouse model, the compound significantly alleviated motor impairment, making it a promising lead compound for further research in PD treatment [138].

Curcumin, a dietary polyphenol, has been studied for its potential treatment against neurodegenerative diseases due to its pharmacological activities and ability to cross the blood–brain barrier. However, its potency and bioavailability are limited due to its physical and metabolic instability. Researchers have attempted to chemically modify curcumin to increase its potency, but one study screened curcumin isoxazole and curcumin pyrazole for their inhibitory potency against α-synuclein aggregation. The results showed that curcumin pyrazole derivatives inhibit α-synuclein aggregation and reduce the associated neurotoxicity [139].

A series of carbazole-based stilbene derivatives were designed by Patel DV et al. [140] to develop multitarget-directed ligands for AD treatment. The compounds were evaluated for anti-AD activities, including cholinesterase inhibition, Aβ aggregation inhibition, and antioxidant and metal chelation properties. The best candidate, (E)-1-(4-(2-(9-ethyl-9H-carbazol-3-yl)vinyl)phenyl)-3-(2-(pyrrolidin-1-yl)ethyl)thiourea), showed good inhibitory activities against AChE and BChE, and significant inhibition of self-mediated Aβ1-42 aggregation.

**Table 2:** Studies showing the potential therapeutic role of synthesized heterocyclic compounds in the treatment and management of AD and PD.

Compounds	Condition	Study Model	Dose and Time	Experimental Assays	Outcomes	Reference
N-Substituted 2-aryloxymethylpyrrolidines	AD	HepG2 and SH-SY5Y cells	0.1–100 µM for 24 h	AChE and BChE inhibition activity; antioxidant activity; molecular modeling; cell viability assay	The compound shows dual BChE/FAAH inhibition activity and a high potential to cross BBB	[135]
Diosgenin–indole derivatives	AD	SH-SY5Y cells ICR mice	0.01–100 mM for 2 h	In vitro: H_2_O_2_- and anti-6-OHDA-induced oxidant assay; anti-Ab assay In vivo: Morris water maze (MWM) test	Effectively improved memory and learning impairments in mice damaged by Aβ	[133]
Indole derivatives	PD	Human HMC3 microglial cells MPTP mice	In vitro: 1–10 µM for 8 h+ 20 h In vivo: 40 mg/kg for 6 weeks (5 times/week)	In vitro: antioxidant assay; In vivo: behavioral tests; neuroinflammation and OS analyses	It reduced MPP+-induced cytotoxicity, reduced NO, IL-1β, IL-6, and TNF-α production, suppressed NLRP3 inflammasome activation, and ameliorated motor deficits, nonmotor depression, and OS in mice	[141]
Indole-3-carbinol	PD	LPS rats	25 and 50 mg/kg for 21 days	Cytokine assay, NF-κB inhibition assay; balance test, open field test (OFT), and MWM	A delay in neurodegeneration of neurons and improvement in motor functions and cognitive function	[142]
Benzothiazole and indole derivatives	PD	M17D-TR/αS-3 K::YFP neuroblastoma cells	5 μM to 40 μM for 24 h	α-Syn inclusion-forming neuroblastoma cell experiment; tau fibril inhibition assay	Inhibit α-syn oligomer activity, but not tau oligomers	[143]
Indanone derivatives	AD and PD	Perphenazine (PPZ)-induced (PD) LPS-induced mice (AD)	PD mice model: 20 mg/kg, p.o.; AD mice model: 250 µg/kg, i.p	Memory assay	Improve cognitive function	[144]
1,4-Dihydropyridine derivatives	NDD	SH-SY5Y; rat hippocampal slices; glia from cerebral cortex of Sprague Dawley rats	Cell line: 1 μM for 24 h; hippocampal slices: 10 µM for 6 h	Neuroprotection studies; anti-inflammatory capacity; GSK-3 inhibitory capacity; voltage-dependent calcium channel blockade assay	Have high antioxidant activity, potent anti-inflammatory capacity, GSK-3 inhibitory capacity, and VDCC antagonist activity; can cross the BBB	[145]
3,5-Diarylpyrazole analogs	AD	MC65 cell lines; adult female Swiss albino mice	Cell line: 1–50 µM; mice: 30 mg/kg	Cholinesterase inhibitory activity and SAR studies; AChE enzyme kinetic assay; behavioral studies (in vivo)	Decreased metal-induced Aβ1-42 aggregation; better spontaneous alternation score and novel arm entries without influencing the locomotor activity	[146]
Curcumin pyrazole and its derivative (N-(3-Nitrophenylpyrazole)	PD	SHSY5Y neuroblastoma cell line	210 µM for 24 h	Determination of cytotoxicity; aggregation assays	Inhibit α-syn aggregation	[139]
CNB-001, a pyrazole derivative	PD	Adult male C57BL/6 mice	24 mg/kg body wt. for 7 days	Behavioral assay; analysis of striatal dopamine and its metabolites	Mitigated motor impairments, reduced behavioral impairments, and significantly reduced striatal dopamine and its metabolite levels in mice while also protecting dopaminergic neurons from MPTP toxicity	[147]
Pyrazolo[3,4-b]quinoline and benzo[b]pyrazolo[4,3-g][1,8]naphthyridine derivatives	AD	10 nM to 1 µM	SH-SY5Y cells	AChE/BuChE inhibitory activity; neuroprotective effect	Protect against rotenone/oligomycin A-induced neuronal death; inhibit AChE/BuChE activity in vitro	[148]
3,4-Dimethyl coumarin scaffold derivatives	AD	SH-SY5Y cells	1 and 10 μM for 24 h	AChE and MAO-B inhibitory assay	Inhibit hAChE and hMAO-B; reduce OS	[149]
Dibenzo[1,4,5]thiadiazepine	NDDs	Human neuroblastoma cell line SH-SY5Y	3 mM for 24 h	Cholinesterase inhibitory activities, cell viability experiments, neuroprotection studies, cytosolic calcium concentration	Shows significant calcium channel modulation activity and is found to be effective in sequestering mitochondrial ROS	[150]
N-Heterocyclic amine	AD	In vitro biochemical assays and FRDA and neuronal (HT-22) cell line	1 μM for 20 min	Antioxidant capacity and amyloid disaggregation	The compound protects amyloid from copper ions and disaggregated amyloid aggregates, with antioxidant activity observed in both cell lines	[151]
3-Amidocoumarin derivatives (coumarins 1–17)	NDDs	Rat cortical neuron culture	100 mm for 24 h	MAO in vitro inhibition; neuronal survival; PAMPA	Cross the BBB and thus exert activity in the CNS; notable neuroprotection from OS	[152]
Ethyl nipecotate (ethyl-piperidine-3-carboxylate) nipecotic acid	AD	Wistar rats	0.15 mmol/kg for 3.5 h	In vitro lipid peroxidation inhibition; AChE inhibition activity	Significant antioxidant potential; GABA reuptake inhibitor; inhibits AChE and reduces rat paw edema	[153]
Pyridine-based hybrids linked to the 1,2,3-triazole unit	Neurological diseases	Outbred mice	50 to 500 mg/kg	Anticonvulsant effect; psychotropic properties; OFT, elevated plus maze, forced swimming, and test for learning and memory	High anticonvulsant and psychotropic properties	[154]
1,2,4-Thiadiazolylnitrones and furoxanylnitrones	NDDs	SH-SY5Y cells	0.05–10 μM for 24 h	PAMPA, antioxidant activity	Compounds exhibit strong free radical scavenger properties and potential therapeutic applications in preventing cell death from OS and damage	[155]
Rosiglitazone	AD	K670N/M671L-transgenic mice overexpressing human hAPP	5 mg/kg for 4 weeks	Object recognition and MWM tests; Aβ plaque deposition assay (ELISA)	Ameliorates memory deficits; rosiglitazone shown to decrease brain Aβ levels and Aβ plaque deposition; reduces p-tau aggregates	[156]
Riluzole	AD	Aβ25-35-induced rat	10 mg/kg/day p.o.	MWM tests; AChE activity and OS marker assay	Improves spatial memory, retention, and recall in MWM and passive avoidance tasks, but is not neutralized by muscarinic or nicotinic receptor antagonists	[157]

In recent experimental work, two novel derivative series acting as hMAO-B inhibitors were developed and evaluated by the researchers. The primary amide group, which is known to be a crucial pharmacophore in the subsequent activity screening and reversible mode of action, has been carefully added to these series, which use benzimidazole as a scaffold. With an IC50 value of 67.3 nM in vitro, 16d is the most effective hMAO-B inhibitor among these substances. Furthermore, 16d demonstrated a favorable safety profile in mouse tests for acute toxicity and cellular damage. Additionally, it demonstrated optimal pharmacokinetic characteristics and blood–brain barrier permeability in vivo, which are crucial requirements for medications that target the CNS. 16d dramatically reduced motor impairment, particularly muscle relaxation and motor coordination, in the MPTP-induced PD mouse model. Being a lead compound, 16d thus has instructional value for further research on its use in the treatment of PD [138].

Based on the location of individual nitrogen atoms arising from various imidazole and pyridine ring annulations, imidazopyridines are classified into four categories. According to the proper nomenclature, all of the scaffolds are classified as imidazopyridines, even if they vary in their structures or even in the number of nitrogen atoms (imidazo[1,2-a]pyridine I and imidazo[4,5-c]pyridine IV). Derivatives of imidazopyridines interact with several biological targets in the CNS, making them a flexible structural template for the development of new CNS modulators [158].

**Table 3:** In vitro studies showing the inhibitory impact of synthetic heterocyclic compounds on major therapeutic enzymatic targets of AD and PD.

Compounds	Condition	Experimental Assays	Outcomes	Reference
6-Amino-substituted imidazo[1,2-b] pyridazines	NDDs	DPPH radical scavenging activity; AChE inhibition assay; molecular docking	Antioxidative/antiparkinsonian agents for important metabolic functions	[159]
4-(Benzylideneamino)- and 4-(benzylamino)-benzenesulfonamide derivatives	AD	AChE activity determination, in vitro inhibition studies, ADMET analysis	Potential inhibitor properties for AChE	[160]
Thiazolyl-pyrazoline derivatives (3a-k)	AD	AChE activity assay, CA activity assay, AChE and CA kinetic analysis	Inhibit AChE and hCA activity	[161]
Isoindole-1,3-dione-substituted sulfonamides		AChE enzyme activity; molecular docking study	Inhibit AChE and hCA activity	[162]
Isoindolines/isoindoline-1,3-diones	AD	Anticholinesterase activity assay; molecular docking	AChE inhibitors	[163]
Imidazo [2,1-B][1,3,4] thiadiazole	AD	BACE1 enzymatic assay; inhibitory activities against AChE and BChE	Superior BACE1 inhibitory activity; potential inhibitory activity against cholinesterase (AChE and BChE)	[164]
1-Hydroxy-2(1H)-pyridinone-based chelators	PD	Modeling methods	Potential to inhibit COMT	[165]
N-substituted pyrazole-derived α-aminophosphonates	AD	Inhibition assay on cholinesterase; evaluation of the cytotoxic activity; antioxidant activity assay	Better AChE inhibitory activity; did not show any cytotoxicity and have promising antioxidant activities against DPPH and H_2_O_2_ scavenging	[166]
Nitrocatechol derivatives of chalcone	PD	MAO-A, MAO-B, and COMT inhibition assay	Potent inhibitors of MAO and COMT	[167]
1,3- Oxazole analogs	AD	AChE and BChE inhibition activity	Ability to inhibit AChE and BChE	[168]
Nitrogen-based novel heterocyclic compounds	AD	AChE and α-glycosidase enzymes were evaluated	Potentially inhibit AChE and α-glycosidase	[124]
Heterocyclic amines (F3S4-m, F2S4-m, and F2S4-p)	AD	BACE1 and AChE inhibition activity and Aβ oligomerization assay	Inhibit Aβ1–42 aggregation and AChE and BACE1 enzyme activities	[169]
6-Benzothiazolyl urea, thiourea, and guanidine derivatives	AD	ABAD’s enzymatic activity	Potent inhibitors of ABAD/17β-HSD10 and potential drugs for AD treatment	[170]

As a heterocyclic aromatic molecule with six members, pyrimidine has nitrogen atoms at positions one and three. This moiety has been used in medicinal chemistry in a variety of ways in recent years. Researchers are drawn to pyrimidine derivatives because of their therapeutic value and adaptable scaffolds [171]. Marketed medications like piribedil, which is used to treat PD, demonstrate their promise for use in NDD medicine. Additionally, a number of molecules containing pyrimidines have been identified as anti-neurodegenerative medicines in preclinical and clinical trials. By inhibiting different enzymes and targets involved in NDDs, the drugs’ activity is explained. Pyrimidine analogs show promise as anti-neurodegenerative drugs. These compounds’ biological evidence supports their potential as multitarget enzyme inhibitors, demonstrating their pharmacological potential by inhibiting targets like ChE, Aβ, MAO-B, KMO, SOD1, Kv, and CX3CR1 that are implicated in several neurodegenerative disorders. Therefore, it is evident from the above that molecules having pyrimidine scaffolds have several uses in the development of drugs to treat NDDs [172].

Several mental illnesses, including anxiety and depression, have etiological relationships with monoamine levels. In theory, a suitable way to evaluate the antidepressant qualities of novel medication candidates is to block MAO [173].

From apixaban, an anticoagulant used to treat and prevent blood clots and stroke, to bixafen, a pyrazole-carboxamide fungicide used to control diseases of rapeseed and cereal plants, the remarkable prevalence of pyrazole scaffolds in a diverse array of bioactive molecules has prompted medicinal and organic chemists to investigate novel approaches in creating pyrazole-containing compounds for various applications [174]. Recently, a study investigated the neuroprotective properties of CNB-001, a novel pyrazole derivative of curcumin and cyclohexyl bisphenol A, in PD. The study found that CNB-001 significantly reduces motor impairments, lowers dopamine levels, and upregulates inflammatory and apoptotic markers. However, co-treatment with CNB-001 attenuated these effects. The results suggest CNB-001’s potential as a therapeutic candidate for PD treatment [147].

Turkan and colleagues synthesized and evaluated the inhibitory effects of pyrazole derivatives (1–8) against the human carbonic anhydrase isoenzymes I and II, as well as the AChE, BChE, and α-glycosidase metabolic enzymes. Their studies revealed that a series of substituted pyrazol-4-yl-diazene derivatives effectively inhibit α-glycosidase, cytosolic hCA I and II, BChE, and AChE. Recently, the inhibition of these metabolic enzymes has emerged as a promising avenue for pharmacologic intervention in a variety of conditions, including NDDs [175].

The pyrazoline (five-membered) heterocyclic ring compound is of great importance pharmaceutically, and its derivatives are of research interest, currently being explored for drug development. Because of their high stability, medicinal chemists have been motivated to experiment with the structure of the ring in a wide range of ways to produce a wide range of pharmacological actions. Pyrazoline rings are found in the architecture of several pharmaceutical products that are sold commercially. The ability of pyrazolines to cure neurodegenerative illnesses is widely established. The NDDs that impact large populations worldwide include psychiatric diseases, AD, and PD [176].

Hitge et al.’s [177] study proposes molecular docking to study potential binding modes and interactions with COMT. The study identifies dual MAO-B/COMT inhibitors by synthesizing chalcone derivatives and converting them to pyrazoline derivatives. The pyrazoline derivatives were found to be more potent than chalcones in inhibiting human MAO and rat COMT. However, these derivatives were weak MAO inhibitors [177].

The goal of another study was to find new dual inhibitors of COMT and MAO, which are involved in the metabolism of dopamine and L-dopa. Chalcone nitrocatechol compounds were created for this aim and assessed as COMT and MAO inhibitors. While nitrocatechol derivatives, such as tolcapone and entacapone, are therapeutically employed COMT inhibitors, the chalcone class of drugs is well known for its strong inhibition of MAO-B. All of the compounds are highly effective in vitro inhibitors of rat liver COMT, according to the data. The most effective inhibitors for the in vitro inhibition of human MAO-B are chalcones, which are less effective as MAO inhibitors. This study suggests a generic method for boosting MAO-B inhibition while maintaining the strong COMT inhibitory activity of this class. It also demonstrates that nitrocatechol derivatives of chalcone may function as COMT and MAO-B inhibitors [167].

Abdelgawad and colleagues produced halogenated chalcones and assessed how well they inhibited MAOs. Compared to MAO-A, all of the synthesized chalcones exhibited stronger and greater inhibitory efficacy against MAO-B. Interestingly, CHB3 and CHF3 inhibited MAO-B more strongly than the reference compounds pargyline and lazabemide. Both compounds inhibited MAO-B reversibly and competitively. According to the findings, CHB3 may be a promising treatment for neurological conditions like PD [178]. Because of its dual inhibitory effects on MAOs and ChEs, the oxygen-containing bicyclic molecule coumarin is frequently employed to create medicines for NDDs [179]. Coumarin–triazole trihybrids with phenylpiperazine, triazole, carbazole, donepezil pharamcophore, and arylisoxazole moieties exhibit good AChE enzyme inhibitory action, representing potential neuroprotective agents for AD treatment [180].

### 3.2. Sulfur-Based Heterocycle Moieties

Many studies on S-containing compounds in the context of AD have been carried out because of the inherent antioxidant potential of S-based heterocycles, and some are presently undergoing clinical trials. Thiazole, which has the formula C3H3NS, is a heterocyclic chemical molecule having a five-membered molecular ring structure. Because of the acidic proton at position C-2, the thiazole ring is extremely reactive and has become a key synthon to produce a range of NCEs (new chemical entities). Numerous new compounds with a broad range of pharmacological actions, including antioxidant, antibacterial, antifungal, antitubercular, diuretic, anti-inflammatory, anticancer, and antipsychotic properties, have been produced by diverse modifications of the thiazole ring at different locations [181]. A diverse range of therapeutic medicines targeting various CNS targets have been developed using thiazoles. Several thiazole derivatives are currently undergoing clinical studies, and thiazole-containing therapeutic molecules are currently being used to treat a variety of CNS illnesses. Thiazole and its analogs have been the subject of numerous studies, which have demonstrated their effectiveness in treating several CNS disorders in both rodent and primate models [182].

CA inhibitors are based on sulfonamide groups; their primary disadvantage is that they nonspecifically block all CA isoforms, which might result in undesirable side effects. Askin et al. (2021) [183] reported an investigation of novel derivatives of imidazole and thiadiazole that do not possess the zinc-binding sulfonamide group for inhibiting human CA I and II isoforms and AChE. Imidazo[2,1-b][1,3,4]thiadiazoles showed low nanomolar inhibitory activity against hCA I, hCA II, and AChE. Compound 9b inhibited hCA I 18-fold more than acetazolamide, while compound 10a selectively targeted hCA II. These compounds could be potential lead compounds for further design [183].

The FDA-approved adenosine A receptor antagonist (anti-PD medication) istradefylline is effective. However, it has been shown that when istradefylline is exposed to direct light or an indoor atmosphere in a diluted solution, its double bond readily transforms into a cis structure. The compound’s series (12 compounds) was created by keeping the xanthine skeleton of istradefylline the same while substituting thiazole orbenzothiazole and other physiologically active heterocyclic compounds for the trans-double bond in order to find more stable adenosine A receptor antagonists with comparable pharmacological efficacy to istradefylline. These compounds were created by a multi-step procedure, and their capacity to prevent the generation of cAMP in cells that overexpress AAR was successfully verified using several characterization approaches. In contrast to istradefylline, the thiazole derivative of istradefylline demonstrated notable activity. Furthermore, benzothiazole derivatives and thiazole compounds with a higher rate of inhibition were molecularly docked and compared to istradefylline. Therefore, this research may serve as a foundation for the logical development of adenosine, a potent receptor antagonist [184].

For more than a century, methylene blue (MB), a tricyclic phenothiazine often referred to as methylthionine hydrochloride, has been used for a variety of medical purposes, including targeting specific cellular targets. MB has been shown to prevent tau aggregation in vitro and has beneficial effects on AD and memory improvement [185]. According to Akour E and colleagues’ research, the interaction between the reduction in and oxidation of tau’s native cysteine residues forms the basis of the process that inhibits tau aggregation. Because MB and its metabolites keep tau in a monomeric disordered shape, they stop filaments and their harmful precursors from forming [186].

Derivatives of fused thiophene are important heterocycles in medicinal chemistry, with fascinating uses in many different domains. They have a wide variety of biological activities including anti-inflammatory, analgesic, and antidepressant activities. Fused thiophene is a potent therapeutic molecule for AD treatment due to its targeting of Aβ aggregation and enzymes including cholinesterases, MAO, and glycogen synthase kinase-3 [187].

In their work, González-Muñoz and colleagues described a novel family of dibenzo[1,4,5]thiadiazepines (1-12) that exhibited an intriguing pharmacological profile in vitro, including antioxidant and neuroprotective qualities, in addition to blocking cytosolic calcium entry. Most of them were anticipated to be CNS-permeable drugs and did not exhibit any deleterious effects. When substances were treated with human neuroblastoma cells 24 h before the addition of toxic stimuli, the cells demonstrated good neuroprotective capabilities against mitochondrial OS, often reaching near-complete protection (>90%). These numbers were lower under co-incubation settings, but certain compounds still exhibited an intriguing degree of neuroprotection—above 50%. Four chosen compounds were discovered to be efficient antioxidants by containing mitochondrial ROS. Additionally, the molecules demonstrated a surprising ability to modulate calcium channels. The fact that dibenzo[1,4,5]thiadiazepine is a little-known structure that is easy to synthesize and has few reported derivatives adds to the interest in these compounds and opens up a new and expansive field of study in medicinal chemistry [150].

Porcal W et al. (2008) synthesized the new compounds 1,2,4-thiadiazolylnitrones and furoxanylnitrones as neuroprotective agents for human neuroblastoma cells. They inhibited oxidative damage and death induced by hydrogen peroxide exposure, demonstrating their potential as neuroprotective agents [155].

### 3.3. Oxygen-Based Heterocycle Moieties

Oxygen-based heterocycles form the core structure of many biologically active molecules as well as U.S. FDA-approved drugs. Moreover, they possess a broad range of biological activities. Often found in remarkably bioactive compounds and a wide range of natural products, oxazole is a biologically active scaffold on which pharmacophores are built to produce powerful, selective medicines. Using nineteen hybrid 1,3-oxazole-based benzoxazole analogs, Hussain and his team assessed their capacity to inhibit AChE and BChE. All analogs showed varied levels of AChE and BChE inhibition in comparison to the conventional donepezil [168]. Opicapone inhibits COMT and is commonly used to manage the symptoms of PD [188].

Flavone derivatives and 1,2,4-oxadiazole have strong anti-inflammatory and antioxidant properties. Based on the impressive anti-inflammatory and antioxidant properties of the 1,2,4-oxadiazole and flavonoid pharmacophores, one researcher created and synthesized a novel series of 3-methyl-8-(3-methyl-1,2,4-oxadiazol-5-yl)-2-phenyl-4H-chromen-4-one derivatives by pharmacophore combination to find highly effective drugs for the treatment of Parkinson’s disease. They then assessed the anti-inflammatory and antioxidant properties of these compounds for the treatment of PD. Their inhibitory actions against ROS and NO release in LPS-induced BV2 microglial cells were used to conduct a preliminary SAR study. The best compound, Flo8, showed the strongest anti-inflammatory and antioxidant properties. By blocking inflammatory and apoptotic signaling pathways, Flo8 prevented neuronal apoptosis, according to both in vitro and in vivo data. Additionally, in vivo research revealed that the chemical Flo8 raised serum dopamine levels and improved behavioral and motor impairments in MPTP-induced PD model mice. All things considered, this study showed that the chemical Flo8 may be a potential treatment for PD [189].

Chalcones are seen as attractive possibilities for the treatment of NDDs, including PD, because of their straightforward structure and advantageous biological characteristics. Chalcones are characterized as promising α-syn imaging probes, enzyme inhibitors (MAO-B, COMT, AChE), antagonists of adenosine A1 and/or A2A receptors, and agents with anti-neuroinflammatory properties (iNOS suppression or Nrf2 signaling activation) [190].

In a recent study by Ceyhun I et al. [191], twelve novel chalcones (2a–l) were synthesized, and their neuroprotective role was studied by investigating the AChE and BchE inhibitory potentials. The synthetic compounds showed notable action against AChE. Additionally, docking modeling showed that these substances interacted with the enzyme active site in a manner akin to donepezil, suggesting they might be powerful medications for the treatment of AD [191].

Sever B and colleagues designed new thiazolyl-pyrazoline (3a-k) compounds to inhibit AChE and hCA activity. The in vitro and in silico results revealed that among 3a to 3k, the most promising derivatives were 3a, 3f, and 3d, with significant effects on AChE, hCA I, and hCA II [161]. Two tolcapone analogs, containing a 1H-pyridinone replacement, have been synthesized by Bergin JCJ et al. (2022) to inhibit COMT and protect neurons from oxidative damage. Measurements of pKa, stability constants, and in silico modeling suggest that these compounds are promising candidates for further evaluation, potentially reducing dopamine in the brain and protecting neurons in PD [165].

Various synthetic compounds currently under clinical trials for AD and PD treatment are listed in Table 4.

## 4. Current Challenges and Future Prospects

L-dopa, dopamine agonists, and MAO-B and COMT inhibitors are the only pharmacological classes that are approved for the treatment of motor-related symptoms of PD, despite all the new research. These treatments primarily act on the dopaminergic neuron system. Although they are also accessible, anticholinergic medications and glutamate antagonists are not frequently utilized in daily practice. Since there is currently no successful therapy approach, alternative approaches need to be looked into. One area of research that has always proven essential to enhancing human health is the hunt for new medicinal possibilities. The largest problem the scientific world is still confronting is drug discovery research. A hybrid approach using multitarget-directed ligands (MTDLs) has been widely used to develop therapeutic agents for AD and PD.

Heterocycles are chemical compounds with at least one element other than carbon in their ring structure and have long been acknowledged as recurring scaffolds in medicinal chemistry because of their importance in drug discovery and development. They are used as appealing building blocks in drug design for neuroprotective drugs and thus, because of their structural diversity and adaptability, open the door to novel treatments and better patient results [192].

In addition to the intricacy of the research, the difficulties encountered in the identification, development, and approval of novel chemical entities are frequently costly and time-consuming [193]. Traditional trial-and-error experiments, which are expensive, time-consuming, and unpredictable, are still used in the development of current formulations. A new field called “computational pharmaceutics” has emerged in the last ten years as a result of the exponential growth of computing power and algorithms. This field combines big data, artificial intelligence, and multi-scale modeling techniques with pharmaceutics, and it has the potential to revolutionize drug delivery. Although molecular modeling is a large subject, the three most popular computer modeling components—molecular docking, MD simulation, and ADMET modeling—have proven essential to the simple identification of leads for experimental in vitro and in vivo testing [193,194,195,196].

## Figures and Tables

**Table 1:** FDA-approved heterocyclic drugs for AD and PD treatment.

Drug	Therapeutic Category	Brand Name	Target	FDA Approval	Application
Brexpiprazole	Atypical antipsychotic	Rexulti	Novel D2 dopamine and serotonin 1A partial agonist	2015	Approved for the treatment of depression, schizophrenia, and agitation associated with dementia due to AD
Donepezil	Parasympathomemetic	Adlarity, Aricept, Namzaric	AChE inhibitor	1996	Used to treat the behavioral and cognitive effects of AD and other types of dementia
Rivastigmine	Parasympathomemetic	Exelon, Nimvastid, Prometax	Inhibits both BChE and AChE	2000	Used to treat mild to moderate dementia in AD and PD
Galantamine	Parasympathomemetic	Razadyne	Competitive inhibitor of AChE	2001	Reduces the severity of dementia in patients with AD
Memantine	NAMD antagonist	Axura, Ebixa, Marixino, Namenda, Namzaric	NMDA receptor antagonist	2013	Blocks the effects of Glu in the brain that lead to neuronal excitability and excessive stimulation in AD
Safinamide	MAO-B inhibitor	Xadago	Selective and reversible inhibition of MAO-B with blockade of voltage-dependent Na^+^ and Ca^2+^ channels and inhibition of Glu release	2017	Neuroprotective and neuro-rescuing effects in PD
Istradefylline	-	Nourianz	Targets adenosine A2A receptors in the basal ganglia	2019	Used to treat reduced GABAergic action and motor control in PD patients
Pimavanserin	Atypical antipsychotic	Nuplazid	Interacts with the serotonin receptors, particularly the 5-HT2A and HT2C receptors	2016	Used to treat PD- associated psychotic symptoms without causing extrapyramidal or worsening motor symptoms
Amantadine	Influenza A M2 protein inhibitor	Gocovri, Osmolex	Releasing dopamine from the nerve endings of the brain cells, and stimulation of norepinephrine response; it also has NMDA receptor antagonistic effects	1973 (for PD)	Used to treat dyskinesia in Parkinson’s patients
Benzatropine	Anticholinergic agent and histamine antagonist	Cogentin	Selective inhibition of dopamine transporters	1996	Used as an adjunct in the therapy of all forms of parkinsonism
Biperiden	Anticholinergic agent	NA	Competitive antagonism of ACh at cholinergic receptors in the corpus striatum	1959	An adjunct in the therapy of all forms of parkinsonism and control of extrapyramidal disorders secondary to neuroleptic drug therapy
Trihexyphenidyl	Anticholinergic agent and muscarinic antagonist	NA	ACh receptor (M1 subtype) antagonist	1949	Anticholinergic activity useful in the treatment of symptoms associated with PD
Carbidopa	AADC inhibitor	Crexont, Dhivy, Duodopa, Duopa, Lodosyn, Parcopa, Rytary, Sinemet, Stalevo	Dopa decarboxylase inhibitor used in combination with levodopa	2014	Symptomatic treatment of idiopathic PD and other conditions associated with parkinsonian symptoms
Entacapone	COMT inhibitor	Comtan, Comtess, Stalevo	Administered concomittantly with levodopa and carbidopa; increased and more sustained plasma levodopa concentrations are reached as compared to the administration of levodopa and a decarboxylase inhibitor	1999	Symptomatic treatment of patients with idiopathic PD
Tolcapone	COMT inhibitor	Tasmar	Inhibits the enzyme COMT used as an adjunct	1998	Helps to improve the symptoms of PD such as trembling, difficulty with movement, stiffness, and other symptoms
Opicapone	COMT inhibitor	Ongentys	COMT inhibitor used as an adjunct	2020	Managing motor and some nonmotor symptoms associated with PD

NA—not available; COMT—catechol-O-methyl-transferase; MAO-B—monoamine oxidase B; NAMD—N-methyl-D-aspartate receptor; ACh—acetylcholine; AChE—acetylcholinesterase; BChE—butyrylcholinesterase; AADC—aromatic L-amino acid decarboxylase; Glu—glutamate.

**Table 4:** Summary of synthetic organic compounds under clinical trials for the management of Alzheimer’s and Parkinson’s.

Drug	IUPAC Name	Compound and Class	Structure	Diseases	Mechanism of Action	Clinical Trial No	Clinical Phase	Sponsor Company
Semagacestat (LY-450139)	(2S)-2-hydroxy-3-methyl-N-[(2S)-1-[[(1S)-3-methyl-2-oxo-4,5-dihydro-1H-3-benzazepin-1-yl]amino]-1-oxopropan-2-yl]butanamide	Organic acids and derivatives Class: carboxylic acids and derivatives	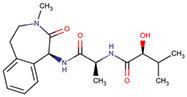	AD	Small-molecule γ-secretase inhibitor	NCT01035138	Phase 3	Eli Lilly and Company
ANAVEX2-73 (Blarcamesine)	[(2,2-diphenyloxolan-3-yl)methyl]dimethylamine	NA	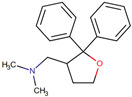	AD	Cognition and function	NCT03790709	Phase 2b/3	Anavex Life Sciences Corp.
Dimebon (Latrepirdine)	5-(2-{2,8-dimethyl-1H,2H,3H,4H,5H-pyrido[4,3-b]indol-5-yl}ethyl)-2-methylpyridine	Organoheterocyclic compounds Class: indoles and derivatives	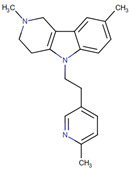	AD	Improves cognition in models of AD	NCT00912288	Phase 3	Pfizer
ALZ- 801 (Valiltramiprosate)	3-[[(2S)-2-amino-3-methylbutanoyl]amino]propane-1-sulfonic acid	NA	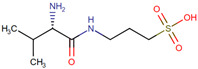	AD	Prevents Aβ42 from forming oligomers	NCT06304883	Phase 3	Alzheon Inc.
Isradipine	3-methyl 5-propan-2-yl 4-(2,1,3-benzoxadiazol-4-yl)-2,6-dimethyl-1,4-dihydropyridine-3,5-dicarboxylate	Organoheterocyclic compounds Class: benzoxadiazoles	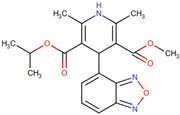	PD	Calcium channel blocker	NCT02168842	Phase 3	University of Rochester
Nilvadipine	3-O-methyl 5-O-propan-2-yl 2-cyano-6-methyl-4-(3-nitrophenyl)-1,4-dihydropyridine-3,5-dicarboxylate	Organoheterocyclic compounds Class: pyridines and derivatives	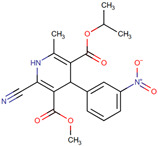		Calcium channel blocker	NCT02017340	Phase 3	Prof Brian Lawlor, St. James’s Hospital, Ireland

NA: not available.
